# CD4+ CAR T-cell expansion is associated with response and therapy related toxicities in patients with B-cell lymphomas

**DOI:** 10.1038/s41409-023-02016-1

**Published:** 2023-06-17

**Authors:** Katharina Baur, Andreas Buser, Lukas T. Jeker, Nina Khanna, Heinz Läubli, Dominik Heim, Jan C. Dirks, Corinne C. Widmer, Thomas Volken, Jakob R. Passweg, Andreas Holbro

**Affiliations:** 1 Division of Hematology, University Hospital Basel, Basel, Switzerland; 2 Innovation Focus on Cell Therapies, University Hospital Basel, Basel, Switzerland; 3 Blood Transfusion Service, Swiss Red Cross, Basel, Switzerland; 4 Department of Biomedicine, University Hospital and University of Basel, Basel, Switzerland; 5 Transplantation Immunology & Nephrology, University Hospital Basel, Basel, Switzerland; 6 Division of Infectious Diseases and Hospital Epidemiology, University and University Hospital of Basel, Basel, Switzerland; 7 Department of Internal Medicine, Division of Oncology, University Hospital Basel, Basel, Switzerland; 8 Diagnostic Hematology, University Hospital, Basel, Switzerland; 9 Department of Health Institute for Public Health, ZHAW Zurich University of Applied Science, Winterthur, Switzerland

**Keywords:** Cancer immunotherapy, B-cell lymphoma

## To the Editor:

Chimeric antigen receptor T-cell (CAR T) therapy is an established and effective option in the treatment of hematological malignancies. Several studies demonstrated that CAR T-cell expansion and persistence correlated with clinical response [1–4]. The two main technological methods for monitoring CAR T-cell expansion include quantitative PCR assays and multiparametric flow cytometry (MFC) [3, 5].

The advantages of MFC include the detection of different CAR T-cell subsets, their activation status, viability, and a short turn-around time [5]. It has been shown that the different composition of CD4+ and CD8+ CAR T-cell populations as well as effector and memory cells influences the efficacy of CAR T-cell therapy [6, 7].

In two patients with chronic lymphocytic leukemia, persistence of CD4+ CAR T-cells was associated with 10-year remission of leukemia [8].

In addition, lymphocyte subsets seem to play a role in the severity of CRS and ICANS. In one study, the production of CAR T-cells without selection of CD8+ central memory T-cells was associated with an increased risk of CRS [9]. Furthermore, severe neurological side effects were associated with higher peak CAR T-cell counts [10] and the expansion and persistence of the CAR T-cells themselves have been discussed as a risk factor for persistent cytopenias [11].

In this retrospective analysis, we aimed to further elucidate the relationship between CAR T-cell expansion and response and the occurrence of complications, paying particular attention to the CD4+ and CD8+ CAR T-cell subsets.

This retrospective, single-center analysis included all patients with relapsed or refractory aggressive B-cell lymphomas who received tisagenlecleucel between February 2020 and December 2021 and for whom CAR T-cell kinetics measured by MFC were available. Median follow-up was 6 months.

Remission status was assessed by PET-CT at 1, 3, and 6 months after CAR T-cell administration.

CRS and ICANS assessment were done according to the ASTCT Consensus Grading [12]. All patients underwent a lymphodepleting chemotherapy with fludarabine and cyclophosphamide or bendamustine (*n* = 1).

All patients gave their written consent for their data to be used for research purposes.

CAR T-cell expansion was measured by MFC from peripheral blood samples using a Biotin labeled soluble CD19 protein and PE-labeled Anti-Biotin antibody (Miltenyi Biotec GmbH, Bergisch Gladbach, Germany). Additional markers were: CD45 Horizon V500, CD3 FITC, CD2 PE-Cy7, CD56 APC, CD4 Horizon V450, CD8 APC-H7 and 7-AAD (BD Biosciences, 2350 Qume Dr, San Jose, CA 95131, United States). The lower limit of CAR T-cell detection was 0.1% of CD3+ T-cells.

MFC was performed on pre-defined time-points (at least every 3 days for the first 2 weeks, then weekly for 3 months, and then once a month until disappearance).

In addition to the maximum expansion of CAR T-cells (peak value), the area under the curve (AUC) was assessed at 28 days, 3 months, and 6 months post-infusion.

Descriptive statics were applied to describe the characteristics of the sample. We employed Generalized Estimation Equation (GEE) models of the Gaussian family with robust standard errors, which adjust for repeated measures of the same patient, to assess associations between CAR T-cell expansion (AUC) and treatment response as well as toxicity. We reported unstandardized coefficients (b) with corresponding 95% confidence interval (95% CI). In order to assess differences between the maximum CAR T-cell expansion and corresponding clinical response, we employed non-parametric Wilcoxon rank-sum tests. Statistical significance was established at *p* < 0.05. We used Stata Version 15.1 (StataCorp, College Station,TX, USA) for all statistical analyses.

Between February 2020 and December 2021, 12 patients diagnosed with relapsed or refractory (after at least two lines) aggressive B-cell lymphomas received tisagenlecleucel.

At the time of CAR-T cell infusion, the median age was 70 years (Table 1 supplementary information).

**Fig. 1:**
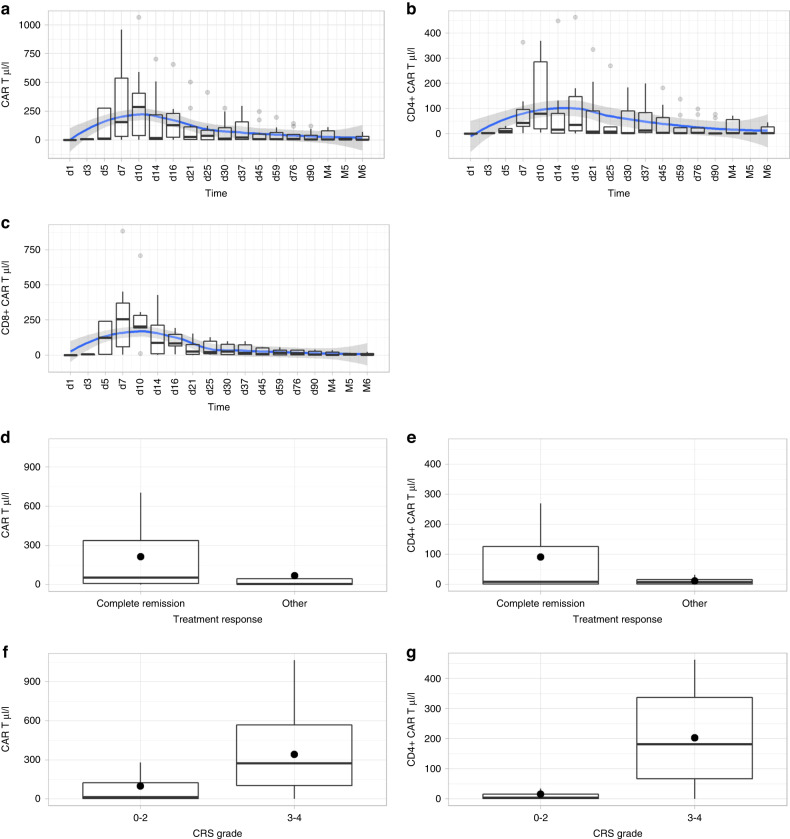
CAR T-cell expansion and association with response and therapy related toxicities. **a**–**c** Course of expansion of total CAR T-cells, CD4 + CAR-T cells, and CD8 + CAR T-cells in the first 6 months after CAR T-cell infusion. **d**–**g** Comparison of CAR T and CD4 + CAR T AUC0-28d in blood of patients with or without CR and CRS at 1 month post transfusion Footnote: Horizontal line represents median, dot represents arithmetic mean.

CAR T-cell expansion increased in the first days reaching a peak value around day 10 (median 285 cells/µl, range 2.3–1064 cells/µl) followed by a decrease during the following weeks (Fig. 1a–c). CD4+ CAR T-cells peak levels were reached around day 10 (median 79.2 cells/µl, range 4.0–368.5 cells/µl) whereas CD8+ CAR T-cells expanded as early as day 7 (median 254.6 cells/µl range 3.5–882 cells/µl).

CAR T-cell expansion was associated with early clinical response. Patients who achieved complete remission (CR) (5 of 12 patients) after 1 month had significantly higher AUC0-28d (b = 165.0 μl/l, 95% CI = 12.0–317.9, *p* = 0.035) (Fig. 1d) and maximum expansion (*p* = 0.017 for the peak value) compared to patients without a CR. At 3 and 6 months, there was no significant difference between response and CAR T-cell expansion within the corresponding time periods. However, patients who were in CR at 3 months had significantly higher CD4+ AUC0-3months (*b* = 61.5 μl/l, 95%CI = 0.5–122.4, *p* = 0.048) compared to patients not in CR. For AUC0-28d we observed a trend that higher CD4+ CAR T-cell levels were associated with CR (b = 86.9 μl/l, 95% CI = −13.6–187.4, *p* = 0.090, borderline significant) (Fig. 1e).

For CD8+ the opposite was found, patients with CR have significantly lower CD8+ AUC0-3months than patients with non-CR (b = 70.7μl/l, 95% CI = 35.6–105.8, *p* = 0.000).

Almost all patients (91.7%) had CRS, with three patients having grade 3 or higher CRS. 25% had ICANS and all were grade 4. All patients with CRS grade 3 or higher had significantly higher AUC0-28d CAR T-cells (b = 246.6 μl/l, 95% CI = 119.1–374.1, *p* = 0.000) and higher AUC0-28d CD4+ CAR T-cell expansion (b = 187.2 μl/l, 95% CI = 91.4–282.9, *p* = 0.000) than patients with lower grade (i.e., ≤grade 2) CRS (Fig. 1f, g). In addition, CRS grade 3 or higher was associated with higher peak values of CD4+ CAR T-cells (*p* = 0.024). No association was observed between expansion or peak levels of CD8+ CAR T-cells and CRS severity. In line with these results, patients with ICANS grade 4 had significantly higher levels of CAR T-cells (b = 330.0 μl/l, 95% CI = 216.2–443.7, *p* = 0.000) and CD4+ CAR T-cells (b = 192.6 μl/l, 95% CI = 90.6–294.7, *p* = 0.000) compared to patients without neurotoxicity.

Higher CAR T-cell levels and especially CD4+ levels have also been significantly associated with higher ferritin levels within the first month (b = 0.02μl/l, 95% CI = 0.01–0.03, *p* = 0.000).

Four of 12 patients showed anemia grade 2 or higher that lasted longer than 1 month. Within the first 6 months after CAR T-cell infusion, higher hemoglobin levels were associated with lower CAR T-cell AUC0-6 months (b = −2.3 μl/l, 95% CI = −4.1–−0.5, *p* = 0.011) and lower CD4+ CAR T-cell AUC0-6 (b = −1.1 μl/l, 95% CI =−2.1–−0.1, *p* = 0.026). No significant association was found between CAR T-cell expansion and platelet and neutrophil count.

Altogether, our results suggest that CAR T-cell expansion, in particular CD4+ CAR T-cells, are associated with better response, but also toxicity.

Several studies have already shown that CAR T-cell expansion and the number of CD4+ CAR T-cells correlates with clinical outcome [1, 2, 4, 8]. However, in our retrospective analysis, we demonstrate—to the best of our knowledge for the first time—that CD4+ CAR T-cells are associated with side effects such as CRS/ICANS and cytopenias.

Although well characterized, the very small number of patients and the retrospective nature of our study are clear limitations. In addition, we determined only CD4+/CD8+ subsets and did not measure activation status of the CAR T-cells and other subsets.

If our results can be prospectively confirmed in larger patient populations, enabling to define clinical meaningful cut-off values regarding postinfusion CAR T-cell proliferation, MFC could be an important and rapid tool for early intervention to prevent severe toxicity and prognostication of clinical outcome. Furthermore, this would allow us to identify a subgroup of patients who would eventually need further anti-lymphoma treatment. In addition, interventions to promote proliferation and persistence of CD4+ CAR T-cells should be investigated.

## Supplementary information

Supplementary table 1

## Data Availability

The datasets used and analyzed during the current study are available from the corresponding author on reasonable request.
