# Supplementary material for: CD4+ CAR T-cell expansion is associated with response and therapy related toxicities in patients with B-cell lymphomas
Source: Bone Marrow Transplant. 2023 Jun 17;58(9):1048–50. doi: 10.1038/s41409-023-02016-1 (PMC10471494; doi:10.1038/s41409-023-02016-1)
Supplement: Supplementary file 1 — Supplementary table 1 [file 41409_2023_2016_MOESM1_ESM.docx]

**Supplementary table 1**

| **Patient demographics, clinical outcomes, and toxicity** | |
| --- | --- |
| **Baseline characteristics** n = 12 (%) | |
| Age at infusion in years, median (range) | 70 years (range, 55-79) |
| Sex | 8 men (66.7), 4 female (33.3) |
| **Histology** | |
| Primary diffuse large B-cell lymphoma | 6 (50) |
| Transformed follicular lymphoma | 5 (41.7) |
| High-grade B-cell lymphoma | 1 (8.3) |
| **Prior therapies** | |
| Lines of prior therapy, median (range) | 2.5 (2-3) |
| History of prior autologous HSCT | 2 (16.7) |
| **Baseline parameters** | |
| Remission status before CAR T-cell infusion | 1 (8.3) CR  6 (50) SD  5 (41.7) PD |
| Hematotox-Score   - low 0-1 - high ≥ 2 | 9 (75)  3 (25) |
| **Lymphodepleting chemotherapy** | |
| Fludarabine and cyclophosphamide | 11 (91.7) |
| Fludarabine and bendamustine | 1 (8.3) |
| **Response** | |
| Remission status after 1 month | 5 (41.7) CR  4 (33.3) PR  2 (16.7) PD  1 (8.3) NA |
| Remission status after 3 months | 7 (58.3) CR  2 (16.7) PR  1 (8.3) PD  2 (16.7) NA |
| Remission status after 6 months | 6 (50) CR  1 (8.3) PR  2 (16.7) PD  3 (25) NA |
| **Toxicity** | |
| CRS   - 0 - grade 1- 2 - grade 3-4 | 1 (8.3)  8 (66.7)  3 (25) |
| ICANS   - 0 - grade ≤ 3 - grade 4 | 9 (75)  0  3 (25) |
| Anemia   - grade ≥ 2/ ≤ 10 g/l (duration ≥ 1 month) | 4 (33.3) |
| Thrombocytopenia   - grade ≥ 2 / ≤ 75 G/l (duration ≥ 1 month) | 9 (75) |
| Neutropenia   - grade ≥ 2/ ≤ 1 G/l (duration≥ 1 month) | 7 (58.3) |

Abbreviations: CR, complete remission; CRS, cytokine release syndrome; HSCT, hematopoietic stem cell transplantation; ICANS, immune effector cell-associated neurotoxicity syndrome; NA, not available; PR, partial remission; PD, progressive disease or relapse; SD, stable disease
